# A comparative study of teaching clinical guideline for prevention of ventilator-associated pneumonia in two ways: face-to-face and workshop training on the knowledge and practice of nurses in the intensive care unit

**Published:** 2015-04

**Authors:** MAJID YAZDANI, GOLNAR SABETIAN, SHAHIN RA'OFI, AMIR ROUDGARI, MONIREH FEIZI

**Affiliations:** 1 Department of Nursing, School of Nursing and Midwifery, Lorestan University of medical sciences, Khoramabad, Iran;; 2 Trauma Research Center, Shiraz University of Medical Sciences, Shiraz, Iran;; 3 Shiraz University of Medical Sciences, Shiraz, Iran;

**Keywords:** pneumonia, nurse, knowledge

## Abstract

**Introduction:**

Ventilator-associated pneumonia (VAP) is one of the most popular nosocomial infections in the intensive care units and the nurse's role in preventing it is very important. The aim of this study was to compare the effect of two methods of face to face training and work- shop clinical guidelines in prevention of VAP.

**Methods:**

In this experimental randomized clinical trial, the knowledge and practice of nurses in ICUs were studied in two groups: face to face training (35 nurses) and workshops (40 nurses) by using clinical guidelines in prevention of VAP in one of the hospitals of Shiraz University of Medical Sciences. The level of knowledge and practice in each group was assessed by self-report questionnaire, knowledge questionnaire and also direct observation of practice, before and after training. Data were analyzed with descriptive statistics, paired t-test, independent t-test, McNemar test, Fisher’s exact, sign and Chi-square test, using SPSS 14.

**Results:**

This study demonstrated that both methods of face to face training and workshop were very effective. The incidence of inappropriate pressure of cuff in the tracheal tubes and tracheostomy tubes was significantly reduced after training (p=0.001). But, by comparison of these two methods and the relationship between the variables revealed that no significant difference was found between the two groups of face to face training and workshop.

**Conclusion:**

Training the nurses is highly effective in preventing VAP, particularly for appropriate cuff pressure, suctioning and disinfecting hands.

## Introduction

Pneumonia refers to the inflammation of the lung parenchyma by biological factors. This complication is the most common respiratory infection. There are two types of pneumonia: community-acquired pneumonia and nosocomial -acquired pneumonia. Ventilator-associated pneumonia (VAP) is a subset of nosocomial pneumonia and the most common infectious complication among ICU patients (1, 2). This complication occurs 48 hours or more after a patient is intubated and connected to the ventilator.

VAP is the second largest American hospital infection (3). It is the second most common nosocomial infection in the intensive care units and the most common in mechanically ventilated patients (4, 5). The clinical pulmonary infection score takes into account clinical, physiological, microbiological, and radiographic evidence to allow a numerical value to predict the presence or absence of VAP (6).

Considering the incidence of VAP in the developed countries between 9 to 27%, many sources in Iran reported that the incidence of VAP in Iran is higher than that of developed countries (7, 8). It has been reported that the VAP mortality rate is between “10%” to “40%” (9). The length of the ICU stay in patients with VAP was 4 to 19 days more than those who were being mechanically ventilated without VAP (10). Hospital costs associated with VAP were estimated about 40,000$ to more than 57,000$ for each case of VAP (11). Therefore, according to the irreversible complications of VAP that include increased patient mortality, increased length of hospital stay and increased hospital costs, prevention of these complications seem essential. The nurses in the ICU due to constant contact with the patient and  performing most of the procedures and guiding others who are in contact with the patient, such as students, health workers and health workers, and help family of the patients have the most important role in the prevention of VAP. Therefore, training of nurses in different ways and testing the effectiveness of the training can be helpful in preventing this complication.

Face-to-face teaching method is a type of direct and presence methods that are applicable with different explanation or practical methods in various sites and diverse opportunities. The advantage of face to face training is the chance to discuss directly with people and urge them to change their behavior. Workshop is a method for solving problems in which individuals (between 25 to 40 people) who are in the same particular scientific or technical field, attend (2). Thus, according to the perilous effects of VAP that have been reported, the present study aimed to compare clinical guidelines on prevention of ventilator-associated pneumonia performed  by either face-to-face and workshop training; also, the effectiveness of these methods on the knowledge and practice of the nurses working in the intensive care unit is considered.

## Methods

In this randomized clinical trial, we studied the nurses' knowledge and performance in two groups: face to face training groups (35 nurses in 2 ICU) and educational workshops (40 nurses in 2 other ICU) by interview and questionnaire. All the hospital ICUs were divided into two groups using simple random sampling. Four ICUs were selected randomly and all of them were the same in the type of the patients and personnel services. This study was done at a hospital affiliated to Shiraz University of Medical Sciences.

The percentages of washing hands, disinfecting hands, wearing treating gloves in contact with the patient, the patients who are not yet suctioned, inappropriate pressure cuff of the endotracheal tube or tracheostomy, the being ready disinfection lotions adjacent patients beds, changing the time of the anti-bacterial filter, and the suitability of the bed angle of the patients for each nurse were under direct observations. The training for the prevention guide of this complication by face to face and workshop training was done in the two groups. After training, in addition to the observations and provision of a questionnaire to assess knowledge, self-report on the questionnaire was also used. The questionnaire used in this study, "questionnaire to assess the knowledge and practice of nurses in prevention of VAP", had been used by Kandeel et al. and Carolyn et al. This questionnaire was localized by adding and removing a few questions and comments of professors (11). Its validity and ratability was approved. The knowledge of the nurses in both groups was re-evaluated.

The results are expressed as descriptive statistics (Mean±SD). The variables of this study were compared between the two groups before and after training by paired t-test, independent t-test, McNemar test, Fisher’s exact test, sign and Chi-square test, using American software SPSS 14. In this study, p≤ 0.05 was considered significant. 

## Results

The incidence of inappropriate pressure of cuff in the tracheal and tracheostomy tubes were significantly reduced after training (p=0.001) and workshops training (p<0/001). The percentage of unsuitable cuff pressure changed from 80% before training to 32.5% after training in the face to face group and from 60% to 15% in the workshop group (Table 1).

**Table 1:** Comparison of cuff pressure in the face to face and workshop training groups

Group	Time statue	Pressure cuff situation	Num	p (comparison between before and after training)
Face to face training	Before	Suitable	8 (20)	p=0.001
		Unsuitable	32 (80)	
	After	Suitable	27 (67.5)	
		Unsuitable	13 (32.5)	
Workshop training	Before	Suitable	16 (40)	p<0.001
		Unsuitable	24 (60)	
	After	Suitable	34 (85)	
		Unsuitable	6 (15)	

(McNemar test)

There were also significant differences in the amount of lack of suction use between before and after training: (p=0.008 for the face to face training group and p=0.002 for the workshop training group). The difference between before and after training was significant (p<0.001). The percentages of this amount changed from 28.5 and 34.2 before to 5.7 after training in face to face and workshop group training, respectively (Table 2). The nurses’ average level of knowledge in both groups increased from 22.36 (before training) to 93.93 (after training), but regarding hand washing and hand rub in contact with the patient before and after training, the frequency was low and inappropriate, so no significant difference was observed. However, both methods of the face to face training and workshop were very effective on nurses’ level of knowledge and practice. For example, in comparison of the two groups before and after training, in the case of pressure of the cuffs, the results were not significant by using Exact Fisher test (p=0.69 before training and p=1 after training).

**Table 2:** Comparison of the amount of the suctions not done before and after training

Group	Time statue	Suctions situation	Num	p-value (comparison between before and after training)
Face to face training	Before	Not done	10 (28.5)	p=0.008
		Done	25 (71.5)	
	After	Not done	2 (5.7)	
		Done	33 (94.3)	
Workshop training	Before	Not done	12 (34.2)	p=0.002
		Done	23 (65.8)	
	After	Not done	2 (5.7)	
		Done	33 (94.3)	

(McNemar test)

 On the other hand, by comparing these two methods and relationship between the variable of the study, no significant difference was found.

## Discussion

In the study conducted by Martin et al. in Brazil which was carried out on the cuff pressure, the mean of the cuffs with inappropriate pressure was 11.6% before training and this rate declined to 6.5% after training; whereas in our study, the mean of the cuffs with unsuitable pressure in both groups was 70% pre-training which decreased to 23.7% after training. Therefore, unsuitable pressure of cuffs in our study was about seven times higher than another study in Brazil. Although washing and disinfecting the hands are the most important factors for infection control and prevention of VAP, the global effectiveness of these factors is reported less than 50%, but in our study the effectiveness of these factors was very low (about 4%). According to this study, our training didn’t make any changes in this regard and this may be due to the fact that nurses work a lot, and have more than one patient in ICU. It is recommended that further studies should be conducted about the effect of this factor in future. Anyway, both the face to face training and workshop training were effective in nursing knowledge, pressure of inappropriate cuff, not suction out the by nurse and, but no significant difference was observed between these two methods.

Observing the nurses without their permissions was the moral problems that we tried to reduce by confidentiality of the nurses’ names and even relevant hospital name.

There were some limitations in this study. The presence of observers could affect the performance of the nurses but they were not very sensitive towards observing them, because the observers had also worked in the same ICU simultaneously. There were other limitations like communication between the groups during the study that we were not able to fully control. 

## Conclusion

Monitoring and evaluating the ICU for the principles of VAP is critical and the role of training in nurses is very effective and inevitable in preventing VAP. Meanwhile, checking the pressure of the cuffs, and washing and disinfecting the hands require special attention.
